# Association Between Nociplastic Pain Criteria and Clinical and Physiological Features in Temporomandibular Disorders: A Cross-Sectional Study

**DOI:** 10.3390/jcm14248967

**Published:** 2025-12-18

**Authors:** Flora Dantony, Daniel Romero-Rodríguez, David Blanco, Carlos Antonio Zárate-Tejero, Carolina Climent-Sanz, Cristina Pérez-Mánen, Natalia Felipe-Spada, Andoni Carrasco-Uribarren

**Affiliations:** 1 Department of Physiotherapy, Faculty of Medicine and Health Sciences, Universitat Internacional de Catalunya, C/Josep Trueta s/n, Sant Cugat del Vallès, 08195 Barcelona, Spain; dromero@uic.es (D.R.-R.); dblanco@uic.es (D.B.); czarate@uic.es (C.A.Z.-T.); ccliment@uic.es (C.C.-S.); cristinaperezmanen@uic.es (C.P.-M.); acarrasco@uic.es (A.C.-U.); 2 Department of TMJ, School of Dentistry, Universitat Internacional de Catalunya, C/Josep Trueta s/n, Sant Cugat del Vallès, 08195 Barcelona, Spain; nfelipe@uic.es

**Keywords:** temporomandibular disorders, nociplastic pain, central sensitization, pressure pain threshold, grip strength

## Abstract

**Background/Objective:** Emerging evidence indicates that temporomandibular disorders (TMD) patients may present features of nociplastic pain (NP), characterized by central sensitization (CS)-related symptoms. This study aims to identify factors associated with CS-related symptoms and pain sensitivity in patients with TMD and NP-related features. **Methods:** In this cross-sectional study, 43 TMD patients were assessed for CS-related symptoms (CSI), proximal, distal, global pressure pain threshold (PPTs, algometer), orofacial pain intensity (VAS), jaw impairment (FAI), maximal isometric strength of four muscle groups (dynamometer), resting heart rate (RHR, chest band), kinesiophobia (TKS-11), physical activity level (IPAQ), anxiety (HADS), and sleep quality (PSQI). Associations were analyzed using linear regression models adjusted for gender, age, and symptoms duration. **Results:** Multivariate analysis showed that higher CSI was significantly associated with greater jaw impairment (Estimate 0.09, 95% CI 0.01 to 0.18) and higher pain intensity (Estimate 0.26, 95% CI 0.14 to 0.38). Lower PPT was significantly associated with lower grip strength (Proximal: Estimate 0.03, 95% CI 0.01 to 0.05; Distal: Estimate 0.07, 95% CI 0.01 to 0.14; Global: Estimate 2.44, 95% CI 0.57 to 4.31), and proximal PPT with higher RHR (Estimate −0.02, 95% CI −0.03 to 0). **Conclusions:** Association was found between CS-related symptoms and pain intensity and jaw impairment, while lower PPTs were related to decreased maximal isometric grip strength and elevated RHR, thus highlighting the need for multimodal treatment in TMD patients.

## 1. Introduction

Temporomandibular disorders (TMD) are complex and multifactorial conditions that often evolve into chronic pain (>3 months), typically presenting as bilateral regional or diffuse discomfort around the ear, jaw, and temporal area [1]. In chronic forms, TMD are increasingly recognized within the nociplastic pain (NP) spectrum, characterized by altered nociceptive processing without clear evidence of tissue damage or somatosensory lesions [2,3].

NP is mediated by mechanisms of central sensitization (CS), involving enhanced responsiveness of central nociceptive neurons and altered sensory integration. These processes are thought to underlie the overlap frequently observed between TMD and other chronic pain conditions such as fibromyalgia, headaches, and neurological disorders [2,4]. Clinically, CS manifests as widespread hyperalgesia, allodynia, fatigue, sleep disturbances, and emotional distress—features often observed in patients with chronic TMD [5].

Although the clinical identification of CS remains debated, quantitative sensory testing, particularly pressure pain threshold (PPT), provides objective markers of hypersensitivity and widespread pain [6]. In parallel, the Central Sensitization Inventory (CSI) has been proposed as a self-reported tool to quantify symptoms associated with CS, including psychological and somatic comorbidities such as anxiety, depression, and fatigue [7,8,9]. However, evidence regarding the relationship between CSI scores and quantitative sensory testing measures remains inconsistent [7,8,9,10], suggesting that both instruments capture distinct, yet complementary, dimensions of NP mechanisms.

Beyond these established tools, identifying additional clinical and physiological features could help better characterize nociplastic manifestations in TMD. Factors such as jaw impairment, muscle strength, cardiorespiratory function, and physical activity level may reflect or exacerbate central sensitization. Altered cortical sensory–motor processing may reduce motor output [10], leading to decreased muscle recruitment and strength. Furthermore, fear of movement and inactivity can worsen cardiorespiratory performance [11], potentially manifesting as increased resting heart rate (RHR) [11]. In chronic or moderate-to-severe TMD, higher emotional distress and lower PPT have been reported [12,13], reinforcing the conceptualization of TMD within the NP continuum [14].

Accordingly, this study aims to analyze the associations between

(i) CS-related symptoms and the clinical and physiological features of TMD, and

(ii) PPT (proximal, distal, and global) and these same features.

Hypotheses:

(i) Higher CS-related symptoms are associated with greater pain intensity, jaw impairment, reduced strength, increased RHR, and psychosocial vulnerability.

(ii) Lower PPT values are associated with greater CS-related symptoms, higher pain intensity, reduced strength, increased RHR, and psychosocial vulnerability.

## 2. Materials and Methods

This study was reported following the Strengthening the Reporting of Observational studies in Epidemiology (STROBE) guidelines [15].

### 2.1. Study Design and Sample Size

This cross-sectional study analyzed data from 43 participants in a randomized controlled trial (NCT06584526) that evaluated the efficacy of aerobic and strength exercise on pain in patients with TMD and features of NP.

The study was conducted in accordance with the Declaration of Helsinki and was approved by the Ethical Research Committee of the Universitat Internacional de Catalunya (FIS-2024-05).

### 2.2. Setting

Participants were recruited via flyers at the Universitat Internacional de Catalunya and were assessed at the University Dental Clinic of the university from September 2024 to June 2025. During this period, 70 potential participants were screened for eligibility. Of these, 9 did not meet the inclusion criteria and 18 declined to participate, resulting in a final sample of 43 patients included in the study. All patients who agreed to participate were provided with written informed consent. Initial screening was performed by a dentist and the physical therapist, who served as the first point of contact to determine eligibility. Eligible participants who were willing to participate were invited to the study. All data were subsequently collected by a separate physical therapist at the Biomechanics and Exercise Physiology Laboratory of the Universitat Internacional de Catalunya.

### 2.3. Participants

The inclusion criteria were as follow: adults over 18 years, with a diagnosis of myogenic or mixed (myogenic and arthrogenic) TMD according to the Diagnostic Criteria for Temporomandibular Disorders (DC/TMD) [16] and reporting neck pain [17]. Among these TMD patients, we further identified and included those presenting features consistent with possible NP, which was assessed according to the IASP clinical criteria and grading system for NP through a Google Forms questionnaire. To meet this criterion, participants had to: (i) report pain of at least 3 months duration; (ii) pain distribution should be regional rather than discrete; (iii) pain cannot entirely be explained by nociceptive or neuropathic mechanisms; (iv) evidence of clinical signs of pain hypersensitivity; and (v) history of pain hypersensitivity in the region of pain (i.e., sensitivity to touch, movement, pressure or heat/cold) and at least one of the defined comorbidities (increased sensitivity to sound, light and/or odors, sleep disturbance with frequent nocturnal awakenings, fatigue or cognitive problems) [4].

The exclusion criteria were: history of temporomandibular or cervical trauma or fracture; previous temporomandibular or cervical surgery; active disease (systemic, rheumatic, metabolic, cardiovascular, neurological, pulmonary, or malignant neoplastic); current orthodontic treatment; occlusal splint; addiction, alcoholism; pregnancy; intake of analgesic or muscle relaxant medication within 48 h before data collection; and physical therapy treatment for the present condition the last 3 months.

### 2.4. Outcomes

#### 2.4.1. CS-Related Symptoms

The Spanish version of the CSI was used to measure CS-related symptoms [18], whose items might be associated with the comorbidities described in the NP criteria [14]. The CSI is a questionnaire of 25 items which assesses the presence of symptoms related to CS, with 5 severity responses ranging from 0 to 4. The total score ranges from 0 to 100 (0–29 = subclinical; 30–39 = mild; 40–49 = moderate; 50–59 = severe; 60–100 = extreme). It demonstrated excellent test–retest reliability (r = 0.91). A cutoff score of 33.5 has been determined to discriminate widespread pain sensitivity and quality of life impairments in chronic musculoskeletal pain [19].

#### 2.4.2. Pain Hypersensitivity

Pain hypersensitivity was measured by determining the Pressure Pain Threshold (PPT) using a digital algometer (Commander^®^ algometer, JTECH Medical, Midvale, Utah), with a surface head of 1 cm^2^, applying a perpendicular force at a constant rate of 0.5 kg/cm^2^/s. Participants were instructed to indicate the point at which the pressure sensation first became painful, at which point the application of pressure was immediately stopped. Assessments were performed bilaterally at the following anatomical sites: temporalis, masseter, sternocleidomastoid, upper trapezius, thenar eminence, patellar tendon, and Achilles tendon. Each site was tested twice, and the mean value was retained for analysis [20].

PPT data were first analyzed separately for each point. Subsequently, mean PPT values were calculated for the proximal trigeminal region (temporalis, masseter, sternocleidomastoid, and upper trapezius), the distal trigeminal region (thenar eminence, patellar tendon, and Achilles tendon), and the global PPT (mean across all points). Calculating regional and global mean PPT values allowed for a more representative segmental and widespread pressure pain sensitivity [21]. The intra-examiner reliability of the algometer has been reported as good, with intraclass correlation coefficients (ICC) ranging from 0.67 to 0.86 [20].

#### 2.4.3. Orofacial Pain Intensity

Orofacial pain intensity was assessed using a 100 mm Visual Analogue Scale (VAS). Participants were asked to indicate the worst orofacial pain they had experienced in the preceding 24 h. The scale ranged from 0 (no pain) to 100 (worst imaginable pain), with participants marking a point along the line corresponding to their perceived pain intensity. The VAS has an excellent test–retest reliability for pain assessment, with an ICC of 0.82 [22].

#### 2.4.4. Jaw Impairment

Jaw impairment was assessed using the Spanish version of the Fonseca Anamnestic Index (FAI) [23]. The FAI consists of 10 items rated on a three-point scale (0 = no, 5 = sometimes, 10 = yes) which assesses both the presence of TMD-related symptoms and their severity. The total score ranges from 0 to 100 (0–20 = no TMD; 20–40 = mild TMD; 45–65 = moderate TMD; 70–100 = severe TMD). Test–retest reliability showed an ICC of 0.937 [23].

#### 2.4.5. Maximal Isometric Strength

Maximal isometric grip strength was measured using a manual dynamometer (J. A. Preston Corporation, Clifton, NJ, USA) with the participant standing and arm fully extended. Maximal isometric strength of the trapezius, quadriceps, and calf muscles was assessed using the Active Force 2 dynamometer (ActivBody, San Diego, CA, USA): trapezius in a seated position, quadriceps seated with hip and knee flexed at 90°, and calf in a seated position on the floor with legs fully extended. A warm-up with the tool consisted of 10 repetitions at moderate force before the actual test. Patients had three seconds to perform the maximal isometric strength. The test was performed twice for each muscle, and the peak mean was calculated. Test–retest reliability showed an ICC of 0.75 to 0.98 [24,25].

#### 2.4.6. Resting Heart Rate

Resting heart rate (RHR) was measured using a Polar H10 (Polar Electro, Kempele, Finland) chest strap connected to the Polar Beat application. Participants were instructed to remain in a supine position for five minutes prior to data collection to ensure resting conditions. The test–retest reliability of this method has been reported as excellent, with an ICC of 0.94 [26].

#### 2.4.7. Fear of Movement (Kinesiophobia)

Fear of movement or activity was assessed using the Spanish version of the Tampa Scale of Kinesiophobia (TSK) [27] The TSK consists of 11 items, each rated on a 4-point Likert scale ranging from “strongly disagree” to “strongly agree” (Likert scale 4 points: 1 = totally disagree; 2 = disagree; 3 = agree; 4 = totally agree). The total score ranges from 11 to 44. The TSK has a good test–retest reliability with an ICC of 0.82 [27].

#### 2.4.8. Physical Activity Level

The Spanish version of the International Physical Activity Questionnaire was used to measure physical activity level [28,29]. The questionnaire measures the frequency (days per week), duration (minutes per day), and intensity (light, moderate, vigorous) of activities performed over the past seven days. Physical activity is reported in Metabolic Equivalent of Task (MET) minutes per week, with participants classified as inactive if less than 600 MET-min/week, minimally active if between 600 and 3000 MET-min/week, and vigorously active if more than 3000 MET-min/week. The IPAQ short form demonstrated good test–retest reliability, with correlation coefficients reaching up to 0.75 [30].

#### 2.4.9. Anxiety and Depression

The Spanish version of the Hospital Anxiety and Depression Scale (HADS) was used to assess anxiety and depression. This scale has been validated in Spanish and consists of two subscales (depression and anxiety), each with 7 items. The score for each subscale can range from 0 to 21. Each item offers four response options, ranging from absence/minimal presence = 0 to maximum presence = 3, with higher scores indicating a greater likelihood of anxiety or depression. This scale has a Cronbach’s alpha coefficient of 0.90 [31].

#### 2.4.10. Sleep Quality

Sleep quality was assessed using the Spanish-validated Pittsburgh Sleep Quality Index (PSQI). The questionnaire consists of 24 items grouped into 7 components, each scored from 0 to 3 (0 = no difficulty; 3 = severe difficulty). The total score ranges from 0 to 21, with a cut-off point of 5 to distinguish ‘good sleepers’ (≤5) from ‘poor sleepers’ (>5). Test–retest reliability showed an ICC of 0.81 [32].

### 2.5. Independent Variables

The independent variables collected were gender, age and duration of symptoms in weeks since the onset of pain.

### 2.6. Bias

As described above, reliable and validated tools were used to collect data. Potential confounders such as age, sex, symptom duration, and psychological factors were collected and considered in the statistical analyses. Efforts were made to ensure that the sample was representative of the population study, to enhance the external validity of the findings. Data analysis was performed by an independent researcher blinded to participants’ clinical status and questionnaire responses.

### 2.7. Statistical Analysis

All analyses were performed using R software (version 4.5.2). Descriptive analyses summarized continuous variables using means ± SD or medians with interquartile ranges (IQRs), depending on data distribution, and categorical variables using counts and percentages.

For the association between CS-related symptoms and study variables, univariable and multivariable linear regression models were fitted, with CSI score as the dependent variable and each explanatory variable included individually as the independent variable. Model coefficients were reported with 95% confidence intervals (CIs) and corresponding *p*-values. Subsequently, a multivariable linear regression analysis was performed to examine the association between CS-related symptoms and clinical and physiological features of TMD: PPT (proximal, distal, global), pain intensity, jaw impairment, maximal isometric strength (quadriceps, trapezius, grip, gastrocnemius), RHR, physical activity level, anxiety and sleep quality. Linear regression models were used for all continuous outcomes (CSI and PPT). The initial model included all candidate explanatory variables. A backward stepwise selection process was applied, using the Bayesian Information Criterion to identify variables for removal, which also helps reduce the risk of overfitting and implicitly addresses potential multicollinearity among related predictors (e.g., CSI, HADS, PSQI, TSK). At each step, a likelihood ratio test was performed to assess whether excluding a variable significantly reduced model fit. This process continued until no further variables could be removed without significant loss of model performance. Model coefficients were reported with 95% CIs and *p*-values. Model validation was performed through residual analysis and calibration.

For the analysis of the association between proximal, distal and global PPTs and clinical and physiological features of TMD (CS-related symptoms, pain intensity, jaw impairment, maximal isometric strength (quadriceps, trapezius, grip, gastrocnemius), RHR, physical activity level, anxiety and sleep quality) the same procedure was applied.

## 3. Results

### 3.1. Descriptive Data

Between September 2024 and June 2025, 43 patients (33 women), with a mean age of 28.3 years (SD 8.1), were recruited for the original randomized controlled trial and were therefore included in the analysis for this study. Median duration of TMD symptoms was 36 weeks (Q1: 18, Q3: 75). The CSI mean score was 40.4 points (SD = 8.36). Mean PPT values varied across test sites, ranging from 1.05 kg/cm^2^ (SD = 0.46) at the sternocleidomastoid muscle to 6.6 kg/cm^2^ (SD = 2.11) at the patellar tendon. Table 1 presents baseline demographics and outcomes.

### 3.2. Univariable and Multivariable Analysis of Variables Associated with CS-Related Symptoms

In univariable analysis, higher CSI scores were significantly associated with higher VAS (*p* < 0.05), FAI (*p* < 0.001), TSK-11 (*p* < 0.05), HADS (*p* < 0.001) and PSQI (*p* < 0.05). However, these associations were no longer present after adjustment for other covariates in the multivariable model, except for VAS (*p* < 0.01) and FAI (*p* < 0.001). These analyses are described in Table 2.

### 3.3. Univariable and Multivariable Analysis of Variables Associated with Mechanical Hypersensitivity

PPT linear regression analyses are described in Table S1. In the univariable analyses, lower distal and global PPT were significantly associated with lower maximal isometric grip strength (*p* < 0.05) and lower maximal isometric upper trapezius strength (*p* < 0.05). However, after adjustment for other covariates in the multivariable model, only the association with maximal isometric grip strength remained significant (*p* < 0.05).

For proximal PPT, univariable analysis showed significant associations with female gender (*p* < 0.05) who presented lower PPT values compared with males, lower maximal isometric grip strength (*p* < 0.001), lower maximal isometric upper trapezius strength (*p* < 0.05), a higher RHR (*p* < 0.05), and a higher TSK-11 (*p* < 0.05). After multivariable adjustment, only associations with maximal isometric grip strength (*p* < 0.05) and RHR (*p* < 0.0) remained significant.

## 4. Discussion

Main findings suggest that higher CS-related symptoms were associated with a higher pain intensity and a greater jaw impairment; while a lower PPT was significantly associated with lower maximal isometric grip strength (proximal, distal, global PPT), and a higher RHR (proximal PPT).

In univariable analyses, higher CSI scores were significantly associated with higher pain intensity, greater jaw impairment (FAI), increased kinesiophobia (TSK), higher levels of anxiety and depression (HADS), and lower sleep quality (PSQI). However, in the multivariable model, only pain intensity and jaw impairment remained significantly associated. This suggests that the other associations may reflect shared variance or confounding effects, and that pain severity and functional limitation may be the most salient in patients with possible nociplastic TMD.

These findings are consistent with previous literature reporting strong associations between CS-related symptoms and increased pain, such as hypersensitivity and allodynia [10,33]. In TMD patients, the chronicity has also been shown to be associated with a higher risk to develop CS [12]. The link between impaired jaw impairment and CS-related symptoms may reflect the influence of stress and parafunctional behaviors, which are known contributors to jaw impairment [16,34]. Furthermore, individuals with greater jaw impairment, as measured by the FAI, have been shown to report higher emotional distress and lower well-being, which might align with a NP phenotype [35].

However, no significant associations were observed between CSI and anxiety, depression, or sleep quality in the multivariable model, despite prior findings showing such relationships [36,37,38]. This divergence may reflect methodological differences, such as higher sample size, or the influence of overlapping variables within multivariate analyses. Indeed, anxiety, depression and sleep quality use to show strong correlations, which makes it difficult to isolate their independent effects [39]. Previous literature shows that subjective sleep quality is closely and bidirectionally related to CS in chronic pain patients. In TMD, insomnia, poor subjective sleep quality as well as objective sleep disturbance such as reduced REM sleep, are associated with higher scores on the CSI, indicating more severe symptoms of central sensitization [40,41,42]. Chronic insufficient sleep can further exacerbate central pain-modulatory dysfunction, increasing vulnerability to CS and chronic pain [43].

These findings collectively emphasize the complexity of TMD, which often involves overlapping biological, psychological, and physiological mechanisms. Clinicians should be aware that CS signs may manifest through diverse pathways, pain intensity, functional limitation, sleep disturbance, or emotional distress, and require an integrative, multidimensional approach to evaluation and management.

CSI was also not associated with physical activity level, in line with previous work reporting similar findings [44], although others have suggested that high-intensity physical activity may exacerbate pain in TMD [45]. The tools used to assess physical activity levels do not provide information on the type or intensity of exercise, which may vary between individuals and explain the effect on pain perception and quality of life [46]. Likewise, no association was found between CSI and kinesiophobia in the adjusted model, despite other studies reporting significant associations [37,38]. This result also contrasts with evidence suggesting that kinesiophobia is associated with increased pain intensity [47] and may contribute to reduced physical activity levels [48]. This discrepancy may be explained by characteristics of the present sample, including relatively young age and milder symptom severity, which could limit the generalizability of these associations.

As for PPT, although higher physical activity can facilitate a descending nociceptive inhibitory mechanism, no significant associations were found with physical activity level. The results contrast with findings from a systematic review and meta-analysis [49], which reported that higher physical activity was related to higher PPT in TMD. As the present cross-sectional study, most of the studies included in the meta-analysis had several proximal and distal PPT, but larger sample size [49].

Lower PPT values were significantly associated with reduced maximal isometric grip strength (proximal, distal and global PPT) and increased RHR (proximal PPT only) in the multivariable analysis. Other factors, such as gender or kinesiophobia, were significant in univariable analyses but did not remain so after adjustment. These findings highlight somatic and physiological correlates of pain hypersensitivity in TMD, which may reflect nociplastic mechanisms [50].

The association between lower maximal isometric grip strength and decreased PPT may reflect central motor inhibition, where the central nervous system could reduce motor output in response to chronic pain [51]. Experimental studies show that muscle nociception does not alter muscle fiber membrane properties or neuromuscular transmission, indicating that motor alterations are mediated by central mechanisms [52]. Nociceptive input can inhibit the primary motor cortex bilaterally, and chronic nociceptive stimulation induces prolonged cortical excitability changes, including motor cortex disinhibition and altered motor responses [53,54]. This may indicate impaired descending pain inhibitory control or central fatigue, linking muscle weakness with altered nociceptive modulation [53]. Similar patterns have been reported in low back pain [55] and fibromyalgia [36], supporting the notion that reduced maximal isometric strength may serve as a physical marker of CS mechanisms. However, in the sample, maximal isometric grip strength was not associated with CSI, reinforcing the notion that the CSI captures a psychometric profile rather than physical function [37].

The link between increased RHR and reduced proximal PPT may reflect autonomic dysregulation. A dysfunction of the central nervous system in pain modulation, as occurs in NP, generally manifests as autonomic alterations, which may interact with CS processes through altered vagal tone and sympathetic overactivation [56]. Dysautonomia has been observed in chronic pain populations and may interact with CS processes through altered vagal tone and sympathetic overactivation [56]. This is consistent with a prior study showing associations between short-term heart rate variability and jaw impairment (FAI), though not with CSI or pain intensity [57]. This could support the use of interventions targeting the autonomic nervous system, such as heart rate variability–guided biofeedback, paced breathing, graded aerobic exercise, and relaxation techniques, alongside conventional pain management [58,59]. Addressing autonomic imbalance might contribute to modulating CS, potentially reducing pain intensity and improving function.

**Limitations and strengths**

It is important to consider several limitations within this study. First, the sample size was small, which limits the generalizability of the findings and warrants cautious interpretation of the results. Moreover, the presence of NP in these patients could not be confirmed, as hypersensitivity was not assessed using a full, validated QST protocol. Although PPT was measured, it was used solely as an objective variable to assess pain sensitivity rather than as a diagnostic tool for NP. Instead, the evaluation relied on self-reported symptoms, which may be less reliable and subject to bias, and only allow for the identification of possible NP rather than a definitive classification. Due to the cross-sectional design of the study, causality cannot be inferred. The observed associations may not reflect directional or mechanistic relationships, and longitudinal studies are needed to clarify whether these factors precede, result from, or progress concurrently with the NP phenotype in TMD.

One of the main strengths of this study lies in its comprehensive, multidimensional assessment, which combines psychometric variables (CSI, pain intensity, jaw impairment), objective physical measures (PPT, maximal isometric grip strength), and autonomic parameters (RHR). The use of these clinical, functional, and physiological measures facilitates straightforward implementation in routine clinical practice, thereby supporting more accurate and practical patient assessment and management. This integrative approach offers a more complete clinical perspective on patients with TMD and possible NP.

**Clinical implications of the findings**

These findings highlight the importance of a multidimensional clinical assessment in patients with TMD and suspected NP. Clinicians should monitor early indicators such as jaw impairment and pain severity to better understand potential progression toward NP. They should also pay attention to signs of autonomic dysregulation, such as elevated RHR, and physical impairments, such as reduced maximal isometric grip strength, inform identification of patient pain phenotypes and guide management strategies.

A multidimensional assessment, combining psychometric tools and physiological measures, enhances the identification of patients with nociplastic features and CS-related symptoms, supporting tailored management strategies that may improve clinical outcomes in TMD.

Future research should aim to better standardize clinical assessment procedures and classification criteria to facilitate more individualized, patient-specific treatment trajectories.

## 5. Conclusions

This study identified clinical variables associated with CS-related symptoms and PPT in patients with TMD and features of NP. CS-related symptoms were mainly associated with pain intensity and jaw impairment, whereas lower PPTs were linked to reduced maximal isometric grip strength and elevated RHR. These findings support the existence of a multidimensional clinical phenotype, combining psychometric and somatic features with underlying CS mechanisms that may manifest as NP. Recognizing this phenotype in clinical practice may facilitate identification and inform mechanism-based management strategies in TMD patients.

## Figures and Tables

**Table 1:** Demographics of baseline data.

Variables	*N* = 43
Gender:	
Men	10 (23.3%)
Women	33 (76.7%)
Age, mean (SD)	28.3 (8.1)
Duration of symptoms (weeks), median (IQR)	36 (18–75)
CSI, score mean (SD)	40.4 (8.4)
PPT (kg/cm^2^), mean (SD):	
PPT proximal	1.82 (0.58)
PPT distal	4.94 (1.60)
PPT global	3.30 (1.01)
PPT temporalis	2.28 (0.82)
PPT masseter	1.63 (0.57)
PPT sternocleidomastoid	1.05 (0.46)
PPT upper trapezius	2.33 (0.95)
PPT thenar eminence	2.97 (1.22)
PPT patellar tendon	6.06 (2.11)
PPT Achilles tendon	5.78 (2.21)
VAS 24 h (mm), mean (SD)	41.7 (24.1)
FAI, score mean (SD)	57.7 (16.8)
Maximal isometric strength (kg), mean (SD):	
Grip	28.9 (7.16)
Upper trapezius	23.1 (9.31)
Quadriceps	26.5 (8.13)
Gastrocnemius	66.7 (10.4)
RHR (bpm), mean (SD)	66.7 (10.4)
TSK-11, score mean (SD)	25.6 (6.7)
IPAQ, score mean (SD)	2347 (1411)
HADS, score mean (SD)	12.1 (5.0)
PSQI score mean (SD)	7.23 (3.3)

Abbreviations: CSI, Central Sensitization Inventory; FAI, Fonseca Anamnestic Index; HADS, Hospital Anxiety and Depression Scale; IPAQ, International Physical Activity Questionnaire; IQR: Interquartile Range; PPT, Pressure Pain Threshold; PSQI, Pittsburgh Sleep Quality Index; SD, standard deviation; RHR: Resting Heart Rate; TSK-11, Tampa Scale of Kinesiophobia (11 items); VAS, Visual Analog Scale.

**Table 2:** Univariable and multivariable linear regression analysis associated with CSI.

Variables	Univariable Analysis			Multivariable Analysis		
	β	95% CI	*p*-Value	Estimate	95% CI	*p*-Value
Gender (women)	2.89	−3.03 to 8.81	0.34	-	-	-
Age	−0.14	−0.46 to 0.17	0.37	-	-	-
Duration of symptoms (weeks)	0.01	−0.01 to 0.03	0.23	-	-	-
PPT (kg/cm^2^):				-	-	-
PPT proximal	−1.43	−5.79 to 2.94	0.53	-	-	-
PPT distal	−0.21	−1.81 to 1.38	0.79	-	-	-
PPT global	−0.47	−2.99 to 2.05	0.72	-	-	-
PPT temporalis	−1.01	−4.1 to 2.07	0.52	-	-	-
PPT masseter	1.81	−3.7 to 7.32	0.87	-	-	-
PPT sternocleidomastoid	1.81	−3.7 to 7.32	0.52	-	-	-
PPT upper trapezius	−1.69	−4.34 to 0.96	0.22	-	-	-
PPT thenar eminence	0.17	−1.93 to 2.26	0.88	-	-	-
PPT patellar tendon	−0.57	−1.76 to 0.63	0.36	-	-	-
PPT Achilles tendon	0.13	−1.03 to 1.29	0.83	-	-	-
VAS 24 h (mm)	0.13	0.03 to 0.23	0.01 *	0.09	0.01 to 0.18	0.037 *
FAI	0.29	0.17 to 0.41	<0.001 *	0.26	0.14 to 0.38	<0.001 *
Maximal isometric strength (kg):						
Grip	0.01	−0.34 to 0.37	0.94	-	-	-
Upper trapezius	0.00	−0.28 to 0.27	0.98	-	-	-
Quadriceps	−0.15	−0.47 to 0.16	0.34	-	-	-
Gastrocnemius	0.04	−0.16 to 0.23	0.72	-	-	-
RHR (bpm)	0.16	−0.09 to 0.4	0.21	-	-	-
TSK-11	0.45	0.09 to 0.81	0.02 *	-	-	-
IPAQ	0.00	0 to 0	0.79	-	-	-
HADS	0.72	0.25 to 0.41	0.00 *	-	-	-
PSQI	0.80	0.06 to 1.53	0.04 *	-	-	-

Abbreviations: CI, Confidence Interval; CSI, Central Sensitization Inventory; FAI, Fonseca Anamnestic Index; HADS, Hospital Anxiety and Depression Scale; IPAQ, International Physical Activity Questionnaire; PPT, Pressure Pain Threshold; PSQI, Pittsburgh Sleep Quality Index; RHR: Resting Heart Rate; TSK-11, Tampa Scale of Kinesiophobia (11 items); VAS, Visual Analog Scale; * *p* < 0.05.

## Data Availability

The data analyzed in this study were derived from the baseline data of the randomized controlled trial registered at ClinicalTrials.gov (NCT06584526). Thus, the datasets are not publicly available but may be obtained from the corresponding author upon request after publication of the randomized controlled trial.

## Supplementary Materials

The following supporting information can be downloaded at: https://www.mdpi.com/article/10.3390/jcm14248967/s1, Table S1: Univariable and multivariable analysis of variables associated with proximal PPT, distal PPT and global PPT.

## Abbreviations

The following abbreviations are used in this manuscript:

CI	Confidence Interval
CS	Central Sensitization
CSI	Central Sensitization Inventory
FAI	Fonseca Anamnestic Index
HADS	Hospital Anxiety and Depression Scale
IPAQ	International Physical Activity Questionnaire
ICC	Intraclass Correlation Coefficient
MET	Metabolic Equivalent of Task
NP	Nociplastic Pain
PPT	Pressure Pain Threshold
PSQI	Pittsburgh Sleep Quality Index
SD	Standard Deviation
RHR	Resting Heart Rate
TMD	Temporomandibular Disorders
TSK-11	Tampa Scale of Kinesiophobia 11-items
VAS	Visual Analogue Scale
