# Supplementary material for: Association Between Nociplastic Pain Criteria and Clinical and Physiological Features in Temporomandibular Disorders: A Cross-Sectional Study
Source: J Clin Med. 2025 Dec 18;14(24):8967. doi: 10.3390/jcm14248967 (PMC12733552; doi:10.3390/jcm14248967)
Supplement: Supplementary file 1 [file jcm-14-08967-s001.zip › jcm-4016234-supplementary.pdf]

Table S1. Univariable and multivariable analysis of variables associated with proximal PPT, distal PPT and global PPT.

	**MEAN PROXIMAL PPT**						**MEAN DISTAL PPT**						**MEAN GLOBAL PPT**					
	**Univariable Analysis**			**Multivariable Analysis**			**Univariable Analysis**			**Multivariable Analysis**			**Univariable Analysis**			**Multivariable Analysis**		
**Variables**	**β**	**95% CI**	**P-value**	**Estimate**	**95% CI**	**P-value**	**β**	**95% CI**	**P-value**	**Estimate**	**95% CI**	**P-value**	**β**	**95% CI**	**P-value**	**Estimate**	**95% CI**	**P-value**
Gender (women)	-0.44	-0.83 to -0.04	**0.04***	-	-	-	-0.64	-1.77 to 0.49	0.27	-	-	-	-0.53	-1.24 to 0.17	0.15	-	-	-
Age	0.01	-0.01 to 0.03	0.52	-	-	-	0.02	-0.04 to 0.08	0.43	-	-	-	0.02	-0.02 to 0.06	0.38	-	-	-
Duration of symptoms (weeks)	0.00	0 to 0	0.50	-	-	-	0.00	0 to 0	0.72	-	-	-	0.00	0 to 0	0.96	-	-	-
CSI	-0.01	-0.03 to 0.01	0.53	-	-	-	-0.01	-0.07 to 0.05	0.79				-0.01	-0.04 to 0.03	0.72	-	-	-
VAS 24h (mm)	0.00	-0.01 to 0.01	0.85	-	-	-	0.00	-0.02 to 0.03	0.65	-	-	-	0.00	-0.01 to 0.01	0.64	-	-	-
FAI	0.00	-0.01 to 0.01	0.48	-	-	-	0.00	-0.03 to 0.03	0.82	-	-	-	0.00	-0.02 to 0.01	0.64	-	-	-
Maximal isometric strength (kg):				-	-	-				-	-	-				-	-	-
Grip	0.04	0.01 to 0.06	**<0.001***	0.03	0.01 to 0.05	**0.013***	0.07	0.01 to 0.14	**0.04***	0.07	0.01 to 0.14	**0.035***	0.04	0 to 0.07	**0.03***	2.44	0.57 to 4.31	**0.014***

Upper trapezius	0.02	0 to 0.04	**0.04***	-	-	-	0.05	0 to 0.1	**0.05***	-	-	-	0.05	0.01 to 0.09	**0.01***	-	-	-
Quadriceps	0.02	0 to 0.04	0.11	-	-	-	0.03	-0.03 to 0.09	0.32	-	-	-	0.02	-0.01 to 0.96	0.23	-	-	-
Gastrocnemius	0.01	-0.01 to 0.02	0.27	-	-	-	0.02	-0.02 to 0.06	0.26	-	-	-	0.01	-0.01 to 0.04	0.25	-	-	-
RHR (bpm)	-0.02	-0.04 to -0.01	**0.01***	-0.02	-0.03 to 0	**0.041***	-0.03	-0.07 to 0.05	0.79	-	-	-	-0.02	-0.05 to 0	0.10	-	-	-
TSK-11	-0.03	-0.05 to 0	**0.04***	-	-	-	-0.05	-0.12 to 0.03	0.22	-	-	-	-0.04	-0.08 to 0.01	0.10	-	-	-
IPAQ	0.0	0 to 0	1.00	-	-	-	0.00	0 to 0	0.50	-	-	-	0.00	0 to 0	0.56	-	-	-
HADS	-0.02	-0.06 to 0.01	0.26	-	-	-	-0.04	-0.14 to 0.06	0.41	-	-	-	-0.03	-0.09 to 0.03	0.35	-	-	-
PSQI	0.00	-0.06 to 0.05	0.98	-	-	-	0.05	-0.1 to 0.2	0.53	-	-	-	0.03	-0.07 to 0.12	0.59	-	-	-

Abbreviations: CI, Confidence Interval; CSI, Central Sensitization Inventory; FAI, Fonseca Anamnestic Index; HADS, Hospital Anxiety and Depression Scale; IPAQ, International Physical Activity Questionnaire; PPT, Pressure Pain Threshold; PSQI, Pittsburgh Sleep Quality Index; RHR: Resting Heart Rate; TSK-11, Tampa Scale of Kinesiophobia (11 items); VAS, Visual Analog Scale; * $p < 0.05$
